# Protest of the Medical Faculty against the Action of the Regents

**Published:** 1868-04

**Authors:** Silas H. Douglass

**Affiliations:** Dean.


**Protest of the Medical Faculty against the Action of the Regents.**

We learn with deep regret the recent action of the State Legislature in attaching the conditions for the appointment of a Professor of Homœopathy to the much needed appropriation recently made for the benefit of the University. Fully appreciating the importance of adding to the pecuniary resources of the institution, we beg most respectfully to present our remonstrance against such an appointment or Professor, and any recognition of him as one of the Medical Faculty, and accompanying it with the following reasons:

First.—It will destroy all good discipline in the medical class. It is utterly impossible for the Faculty to control the conduct of a class of over five hundred students or young men of an average age of over twenty-five, assembled in one class, except that they are actuated by motives of respect to the professors. A Professor of Homœopathy could never command that respect essential to good discipline. He would be inevitably subjected to such insults from the regular students as would render it practically impossible for him to continue his lectures, while on the other hand the regular professors would be equally insulted by the Homœopathic students. Thus strife and ill-feeling would be engendered between the two classes of students that would speedily terminate in uncontrollable riot and disorder. This condition of things is based upon the assumption that the two classes of students assembled in respectable numbers, an assumption which we consider as scarcely warrantable.

Second.—It will seriously if not fatally reduce the number of students. We are dependent upon the good will and good opinion of the medical profession for our students, as the advice and control of the preceptor is certain to give direction to the student. The medical professors regard the practice of Homœopathy, or any other exclusive system, as a species of dishonest quackery, unworthy of their recognition and support; and any affiliation with or countenance of it, they would regard as dishonorable in the extreme. We could no more command the respect or secure the patronage of the medical profession with such an amalgamation in our Faculty than a Presbyterian Theological School could secure students in Theology and instruct them in peace and quiet with a Professor of Mormonism or Infidelity lecturing in its halls as a recognized member of its Faculty.

Third.—It would destroy the good name and standing of the Medical College in the country. An affiliation with Homœopathy would be a violation of the code of medical ethics of the American Medical Association; a highly respectable body, representing the sentiments of the profession in this country. This written code of medical ethics is law unto the profession over the entire Union, and this violation would inevitably lead to the expulsion of the Faculty and the graduates of the institution from every medical association or society in the country. We, the Faculty and graduates, would be outcasts from the profession, and our diplomas would not be worth the parchment upon which they are printed, as passports to admission to the medical profession. Our certificates of attendance would be worthless to the student desirous of attending lectures in another college.

Finally, we feel called upon in duty to ourselves, to the medical profession, and to the department in which we have so long labored with great pride and satisfaction, to express to your honorable board our unqualified conviction that the creation of a chair of Homœopathy in the medical department of the University, and compelling the Medical Faculty to associate with the professor on terms of equality, or on any terms, will deprive the department of the support and sympathy of the profession; will cause its professors to be rejected and expelled in disgrace from every medical association or society in the country; will leave it unrecognized among the medical colleges of the world, and most certainly terminate in the complete destruction of the medical college as a regular school of medicine.

If the substitution of a school, held by an old, honorable and highly eclectic profession, as having its origin in a myth and a fiction, and as being guided and controlled by principles of dishonorable and disgraceful quackery, for the most flourishing Medical College in the country; if the loss of over five hundred good and earnest students, and fees amounting to over $13,000, is a fair and just equivalent for the $15,000 derived from the State, then the hard and earnest work of seventeen of the best years of our lives, and all the wholesome influences upon the medical profession that have been and are being created, are made a sacrifice by the most hasty, inconsiderate and unconsciously injurious legislative action that has ever been made on the subject of public education.

Respectfully submitted in behalf of the Medical Faculty.

SILAS H. DOUGLASS, Dean.

Medical Department, March, 1867.

